# MicroRNA-1258 Inhibits the Proliferation and Migration of Human Colorectal Cancer Cells through Suppressing CKS1B Expression

**DOI:** 10.3390/genes10110912

**Published:** 2019-11-08

**Authors:** Jin-Seong Hwang, Eun-Jeong Jeong, Jinhyeon Choi, Yeo-Jin Lee, Eunsun Jung, Seon-Kyu Kim, Jeong-Ki Min, Tae-Su Han, Jang-Seong Kim

**Affiliations:** 1 Biotherapeutics Translational Research Center, Division of Biomedical Science, Korea Research Institute of Bioscience and Biotechnology, Daejeon 34141, Korea; hjinsung@kribb.re.kr (J.-S.H.); ej0314@kribb.re.kr (E.-J.J.); cluke@kribb.re.kr (J.C.); leeyeoja@kribb.re.kr (Y.-J.L.); silverline09@kribb.re.kr (E.J.); jekmin@kribb.re.kr (J.-K.M.); 2 Department of Functional Genomics, University of Science and Technology, Daejeon 34141, Korea; 3 Department of Biological Science, College of Natural Sciences, Wonkwang University, Iksan 570-450, Korea; 4 Personalized Genomic Medicine Research Center, Division of Biomedical Science, Korea Research Institute of Bioscience and Biotechnology, Daejeon 34141, Korea; seonkyu@kribb.re.kr

**Keywords:** colorectal cancer, CKS1B, miR-1258

## Abstract

Increasing evidence has demonstrated that increased expression of cyclin-dependent kinase regulatory subunit 1B (CKS1B) is associated with the pathogenesis of many human cancers, including colorectal cancer (CRC). However, the regulatory mechanisms underlying the expression of CKS1B in CRC are not completely understood. Here, we investigate the role played by microRNAs in the expression of CKS1B and carcinogenesis in CRC. Among the six microRNAs predicted to target CKS1B gene expression, only miR-1258 was revealed to downregulate CKS1B expression through binding to its 3’-UTR region, as ectopic miR-1258 expression suppressed CKS1B expression and *vice versa*. In CRC, miR-1258 expression also decreased cell proliferation and migration in vitro and tumor growth in vivo, similar to cells with silenced CKS1B expression. Considering the highly increased levels of CKS1B and decreased expression of miR-1258 in tumors from CRC patients, these findings suggest that miR-1258 may play tumor-suppressive roles by targeting CKS1B expression in CRC. However, the therapeutic significance of these findings should be evaluated in clinical settings.

## 1. Introduction

Colorectal cancer (CRC) is the second most commonly diagnosed cancer, and there are 145,600 new cases and 51,020 deaths in the United States in 2019 [1]. CRC progression is multistage and involves various genetic modifications and alterations of molecular signaling pathways [2]. Although CRC diagnosis and treatment have improved patient survival because of the development of surgical techniques and efficient anticancer drugs, the treatment failure rate remains high due to metastasis or multiple chemotherapeutic drug resistance [3,4]. Accordingly, identifying novel genes critical for the carcinogenesis and disease progression of CRC and understanding their molecular mechanisms are required to improve therapeutic efficacies in the treatment of CRC patients.

CDC28 protein kinase regulatory subunit 1B (CKS1B) is a member of the conserved cyclin kinase subunit 1 (CKS1) protein family that interacts with cyclin-dependent kinases (CDKs) and plays important roles in cell cycling [5,6]. CKS1B was identified as an accessory protein to the SKP1-CUL1-F-box (SCF) ubiquitin ligase complex, and Cks1 increased the interaction between SKP2 and p27^Kip1^. CKS1 knockout mouse studies showed the role of Cks1 in the cell cycle; these mice had a small body size due to a lack of proliferation and degradation of the CDK inhibitor p27^Kip1^ [7,8]. Increasing evidence from next-generation sequencing (NGS) data has demonstrated CKS1B upregulation in various cancers, and its overexpression was associated with poor prognosis and high aggressiveness in hepatocellular carcinoma and multiple myeloma [6,9,10].

MicroRNAs (miRNAs) are conserved, short noncoding RNAs that regulate the expression of multiple target messenger RNAs (mRNAs) at the posttranscriptional level, typically through binding to the 3’ untranslated region (UTR) [11]. The mature form of miRNAs affects the stability of the target mRNAs. Partial complementary binding between miRNAs and target genes leads to transcriptional suppression, whereas complete complementary binding induces endonucelolytic cleavage [12]. Recent reports have shown that over 60% of genes have preserved sequences that pair with miRNAs, indicating that miRNAs are widely used to regulate various genes in vivo [13]. miRNAs have functions that are involved in diverse biological processes, such as development, cell cycle regulation, cell proliferation, migration, differentiation, and apoptosis [14,15]. However, miRNA dysregulation may result in numerous diseases, including developmental disorders and neurological diseases, as well as cancer. In particular, miRNAs involved in tumor development have been indicated by the identification of tumor-associated miRNAs via integrated analysis of miRNA–mRNA profiles by RNA sequencing techniques [16,17,18]. The expression of miRNAs can be regulated in part by epigenetic factors such as histone modification and DNA methylation, and miRNA dysregulation via disruption of the balance between miRNAs and their target genes has been reported in cancer development [19,20].

In the current study, we investigated the roles of microRNAs in regulating CKS1B gene expression in CRC. Here, we present experimental evidence showing that miR-1258 plays a tumor-suppressive role by directly regulating the expression of the oncogenic CKS1B gene in CRC.

## 2. Materials and Methods 

### 2.1. Cell Culture

The human CRC cell lines HT29 and HEK293 were obtained from the Korean Cell Line Bank (KCLB, Seoul, Korea), and KM12SM-luc was provided by The University of Texas MD Anderson Cancer Center. HT29, KM12SM-luc, and HEK293 were maintained in DMEM (Welgene, Gyeongsan, Korea) with 10% fetal bovine serum (RMBIO, Missoula, MT, USA) and a 1% antibiotic–antimycotic solution (Thermo Fisher Scientific, Waltham, MA, USA).

### 2.2. Gene Expression Analysis

Total RNA was extracted from cultured cells and xenograft tumor tissues using Nucleosol (Macherey-Nagel Co., Düren, German) according to the manufacturer’s instructions. Total RNA was reverse-transcribed to cDNA using a cDNA synthesis kit (Takara Bio Inc., Shiga, Japan), and quantitative reverse-transcription PCR (qRT-PCR) was performed using SYBR Green (Applied Biosystems, Foster City, CA, USA) with StepOne Plus (Applied Biosystems). The qRT-PCR primers are shown in Table 1. The qRT-PCR conditions were initial denaturation for 10 min at 95 °C, 40 cycles of denaturation for 15 sec at 95 °C, and 1 min of annealing/extension at 60 °C. The expression of miR-1258 was analyzed using an HB miR Multi Assay Kit™ System I (Heimbiotek, Seongnam, Korea). The expression level of miR-1258 was normalized to that of RNU6B.

### 2.3. Protein Expression

Cells were harvested in RIPA buffer with phosphatase and protease inhibitors (Thermo Fisher Scientific). Then, the cell lysates were centrifuged at 12,000 rpm for 30 min. The protein concentration was measured using a Bicinchoninic acid (BCA) kit (Thermo Fisher Scientific). Western immunoblotting was performed using anti-CKS1B (Invitrogen, Waltham, MA, USA) and anti-beta-actin (Abclon Inc., Seoul, Korea) antibodies. The band sizes were quantitatively measured using ImageJ software.

### 2.4. Transfection of miR-1258 Mimic, miR-1258 Inhibitor, Stable miR-1258 Expression Vector and CKS1B siRNA

To transiently induce or inhibit miR-1258 expression, a miR-1258 mimic or inhibitor (Bioneer, Daejeon, Korea) was used to transfect CRC cells. To establish a stable miR-1258-expressing cell line, the full-length coding region of miR-1258 cDNA was amplified and cloned into the pmR-ZsGreen1 vector (Takara Bio Inc.). The miR-1258 cloning primers are shown in Table 1. For the selection of miR-1258 expressing cells, 10 μg/mL geneticin (Thermo Fisher Scientific) was added to the medium, and green fluorescence protein (GFP)-positive cells were sorted with a Fluorescence-activated cell sorting (FACS) sorter. To reduce CKS1B levels, CRC cells were transfected with CKS1B siRNA (Dharmacon, Lafayette, CO, USA) using Lipofectamine RNAi Max (Invitrogen), following the manufacturer’s instructions.

### 2.5. Cell Proliferation and Migration Assays

Cell proliferation was evaluated using a Cell Counting Kit-8 assay (Dojindo Laboratories, Kumamoto, Japan) as previously described [21]. First, 1 × 10^3^ cells were seeded in a 96-well plate, and siRNA, miRNA mimic or miRNA inhibitor was transfected. Cell proliferation was measured at a reference wavelength of 450 nm. To perform the cell migration assays, mimic, inhibitor or siRNA was transfected into CRC cells. After 24 h, 3 × 10^5^ cells were seeded in 24-transwell plates (Corning Inc., New York, NY, USA). After 18 h, the cells were fixed and stained with a 0.1% crystal violet solution (Biosesang, Seongnam, Korea).

### 2.6. Luciferase Reporter Assay

Sense and antisense oligomers, which included the miR-1258 binding sequences, were cloned into the pmiRGlo-Dual luciferase vector (Promega, Fitchburg, WI, USA). Wild-type and mutant oligomers are shown in Table 1. The luciferase reporter vector and mi-1258 mimic or negative control (NC) mimic were transfected into HEK293 cells, and luciferase activity was measured as described previously [17].

### 2.7. Xenograft Mouse Model

To establish a xenograft mouse model, KM12SM-luc cells stably expressing miR-1258 were implanted into the flank of five-week-old female Balb/c-nu mice (Narabio, Seoul, Korea). Tumor size was measured with digimatic calipers at specified time intervals. The animal experiments were approved by the Committee on Animal Experimentation of the Korea Research Institute of Bioscience and Biotechnology.

### 2.8. Statistical Analysis

The Student *t*-test and the Kruskal–Wallis test were used to analyze miRNA and CKS1B expression, and a two-way ANOVA was used to analyze mouse tumor volumes. The Cancer Genome Atlas (TCGA) data set for colon adenocarcinoma (COAD) was downloaded from UCSC Xena (https://xena.ucsc.edu/) and used to analyze the expression level of CKS1B and miR-1258. Statistical significance was determined by the Student *t*-test. The data are presented as the mean ± S.D., and all statistical analyses were conducted using GraphPad Prism version 5.0 (GraphPad Software, San Diego, CA, USA).

## 3. Results

### 3.1. miR-1258 Negatively Regulates CKS1B Expression, Which Is Highly Upregulated in CRC Tumor Tissues

**Figure 1:**
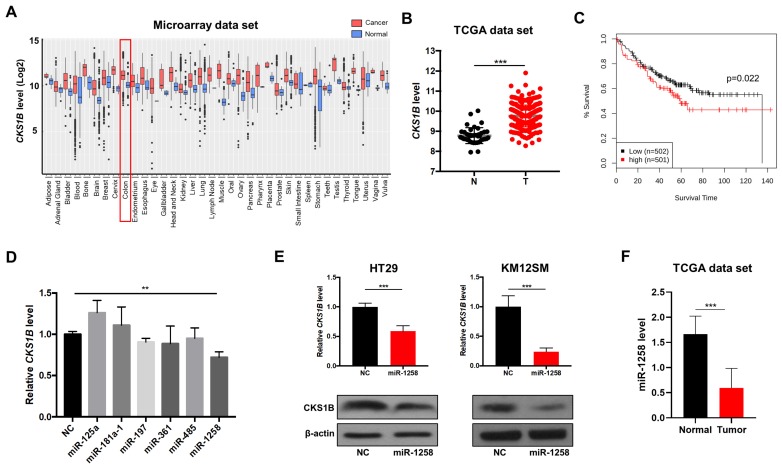
Upregulation of CKS1B expression and its targeting miRNA in colorectal cancer (CRC). (**A**) The expression level of CKS1B in normal (**blue**) and cancer (**red**) tissues from the GENT2 data set. (**B**) Expression of CKS1B in human colon cancer samples (T) and normal samples (N) from the colon adenocarcinoma (COAD) The Cancer Genome Atlas (TCGA) data set. (**C**) Survival analysis for CRC using the GENT2 data set. (**D**) Relative expression level of CKS1B in HT29 cells transfected with negative control (NC) mimic or miR-125a, miR-181a-1, miR-197, miR-361, miR-485 or miR-1258 mimic according to qRT-PCR. (**E**) CKS1B mRNA and protein expression levels in HT29 and KM12SM cells transfected with NC mimic or miR-1258 mimic. (**F**) Expression level of miR-1258 in human colon cancer samples and normal samples from the COAD TCGA data set. ** *p* < 0.01, *** *p* < 0.001.

Previously, it was demonstrated that upregulation of the oncogenic protein CKS1B promotes cell growth, invasion, metastasis, and chemoresistance and that CKS1B expression is dysregulated in various cancer types [6,9]. However, its role in CRC has not been clearly established to date. Accordingly, when we analyzed CKS1B expression levels in various cancer types using the GENT2 public data repository (http://gent2.appex.kr/gent2/), the CKS1B expression level was significantly increased in colon, bone, brain, cervical, liver, lung, ovarian, and pancreatic cancer samples compared to normal tissue samples (Figure 1A). Consistently, increased CKS1B levels in tumor samples were also observed in other human cancer data sets, such as the TCGA cohort (Figure 1B). Moreover, high expression of CKS1B significantly reduced the overall survival of CRC patients (Figure 1C), suggesting that CKS1B upregulation may be associated with the disease progression of CRC.

Next, to understand the regulatory role of miRNAs in CKS1B expression in CRC, we screened CKS1B-targeting microRNAs (miRNAs) through bioinformatics approaches with TargetScan7.2 and microRNA.org. In silico analysis predicted several CKS1B-targeting miRNAs, such as miR-125a, miR-181a-1, miR-197, miR-361, miR-485, and miR-1258. To validate the prediction results, miRNA mimics for each of the above miRNAs were used to treat HT29 colon cancer cells, and quantitative RT-PCR was performed to measure CKS1B levels. Among these miRNAs, only the miR-1258 mimic suppressed CKS1B expression at the mRNA (Figure 1D) and protein (Figure 1E) levels in CRC cells compared to cells treated with negative control (NC) mimic. In addition, miR-1258 downregulation was observed in tumor tissues from CRC patients after gene expression analysis using the TCGA data set (Figure 1F). Taken together, these results indicate that miR-1258 may play tumor-suppressive roles in CRC through negatively regulating oncogenic CKS1B gene expression.

### 3.2. CKS1B Is Directly Regulated by miR-1258

**Figure 2:**
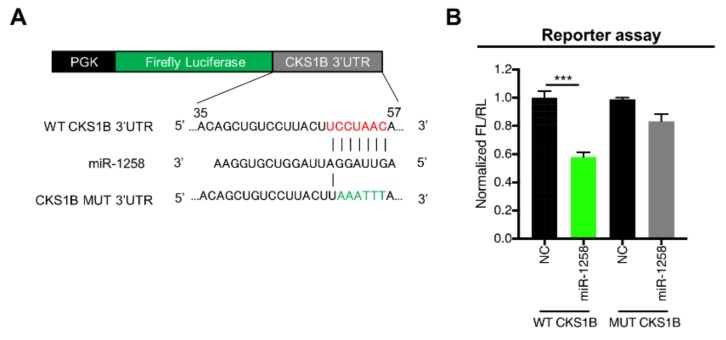
CKS1B is a direct target gene of miR-1258. (**A**) Construction of a dual luciferase reporter vector including normal seed match sequences (WT) or mutant sequences (MUT) of the miR-1258 binding site in the CKS1B 3’-UTR. (**B**) Luciferase reporter assay of the 3’-UTR region of CKS1B in HEK293 cells transfected with miR-1258 mimic and the luciferase reporter vector. All data are presented as the mean ± S.D. of triplicate experiments. *** *p* < 0.001.

To determine whether miR-1258 directly regulates CKS1B gene expression, a luciferase reporter assay was performed using a pmiRGlo-Dual luciferase reporter vector (Figure 2A) containing the wild-type (WT) or mutant (MUT) miR-1258-binding sequences in the 3’-UTR of the CKS1B gene. The miR-1258 mimic and reporter vector were then cotransfected into HEK293 cells. In cells containing WT miR-1258-binding sequences in the 3’-UTR of the CKS1B gene, luciferase activity was significantly lower in cells treated with the miR-1258 mimic than in cells treated with the NC mimic. In cells with MUT miR-1258-binding sequences, however, no difference was observed in luciferase activity between cells treated with NC or miR-1258 mimic (Figure 2B). Together, these results indicate that miR-1258 directly regulates CKS1B expression through the binding sequence in the CKS1B 3’-UTR.

### 3.3. miR-1258 Inhibited Cell Proliferation, Motility and Tumorigenicity

**Figure 3:**
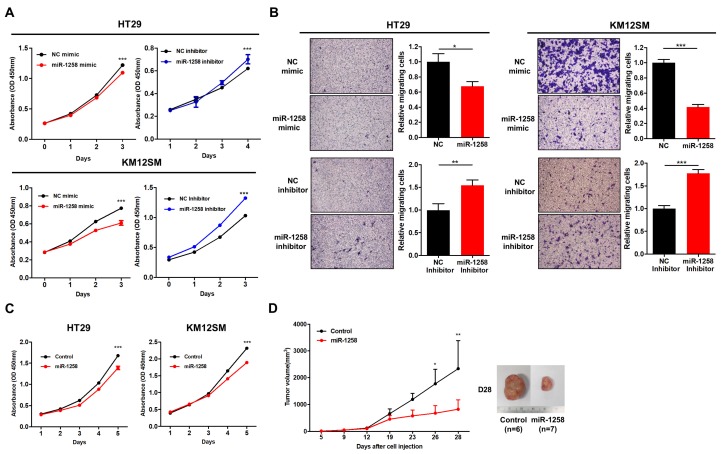
miR-1258 inhibits cell proliferation, migration and tumorigenicity in CRC cells. (**A**) Growth curves of HT29 and KM12SM cells transfected with NC mimic or miR-1258 mimic (left) and NC inhibitor or miR-1258 inhibitor (right). (**B**) Cell migration assay for HT29 and KM12SM cells transfected with NC mimic or miR-1258 mimic and NC inhibitor or miR-1258 inhibitor. (**C**) Cell proliferation assay of stable miR-1258-expressing HT29 (left) and KM12SM (right) cells. (**D**) Tumor volumes for the xenograft mouse model using miR-1258-overexpressing KM12SM cells. * *p* < 0.05, ** *p* < 0.01, *** *p* < 0.001.

As the first step in determining the biological function of miR-1258, a cell proliferation assay was performed using human CRC cell lines. Compared to NC treatment, miR-1258 mimic treatment significantly decreased cell growth, whereas miR-1258 inhibitor treatment increased cell proliferation in both HT29 and KM12SM cell lines in vitro (Figure 3A). Next, we performed a transwell migration assay to investigate the effects of miR-1258 on cell mobility. miR-1258 mimic treatment significantly reduced the number of migrated cells, whereas miR-1258 inhibitor treatment promoted CRC cell motility in vitro (Figure 3B).

To assess the effects of miR-1258 on tumor cell growth both in vitro and in vivo, we established HT29 and KM12SM clones with stable miR-1258 overexpression for use in in vitro cell proliferation assays and for implantation into mice to monitor tumor growth. Compared to the control conditions, stable miR-1258 expression in HT29 and KM12SM cells significantly suppressed cell growth in vitro (Figure 3C) and significantly decreased tumor growth in vivo (Figure 3D). Taken together, these results indicate that miR-1258 may play tumor-suppressive roles in CRC.

### 3.4. CKS1B Knockdown Suppressed CRC Cell Proliferation and Migration 

**Figure 4:**
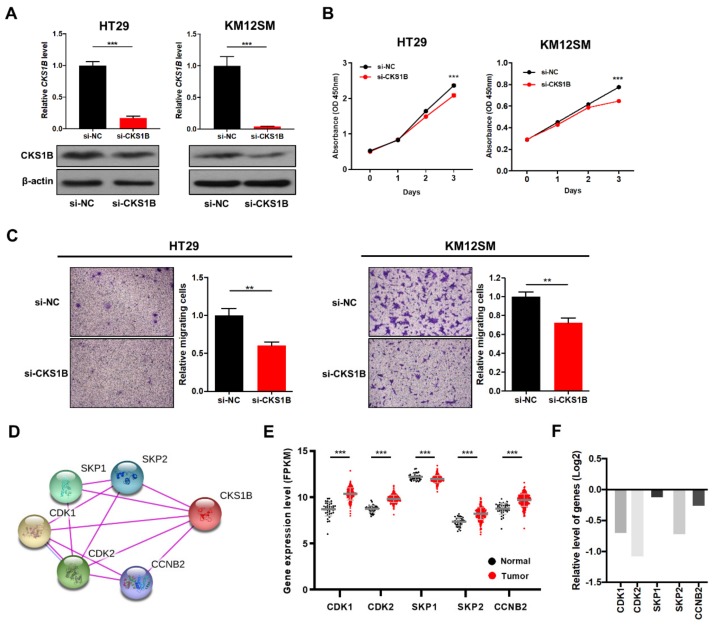
CKS1B knockdown decreases cell growth and migration ability in CRC cells. (**A**) CKS1B mRNA (**top**) and protein (**bottom**) expression levels in HT29 and KM12SM cells transfected with negative control (si-NC) or CKS1B (si-CKS1B) siRNA. (**B**) Growth curves for HT29 and KM12SM cells transfected with CKS1B siRNA. (**C**) Cell migration assay for HT29 and KM12SM cells transfected with CKS1B siRNA. (**D**) Analysis of CKS1B interaction genes with the STRING analysis tool. CKS1B directly interacts with the SKP1, SKP2, CDK1, CDK2, and CCNB2 genes. (**E**) Expression levels of CKS1B-related genes from the TCGA data set. (**F**) Relative expression of genes in sh-CKS1B cells compared to sh-control cells. **p < 0.01, ***p < 0.001.

Based on the findings that miR-1258 negatively regulates CKS1B expression and suppresses CRC cell proliferation and migration, we determined whether the biological activities described above resulted from miR-1258-mediated CKS1B downregulation. We transfected HT29 and KM12SM human CRC cells with CKS1B siRNA and observed that CKS1B mRNA and protein levels were significantly decreased (Figure 4A). Next, cell proliferation and transwell migration assays were performed, and suppressing CKS1B expression significantly reduced the cell growth and migratory abilities of CRC cells (Figure 4B,C), as was observed in miR-1258-treated CRC cells.

We next analyzed functional CKS1B interaction genes with experimental validation sources using the STRING analysis tool (https://string-db.org/). The results showed that CKS1B was connected with cell cycle- and ubiquitin-related genes, including cyclin-dependent kinase (CDK1 and CDK2), S-phase kinase-associated protein (SKP1 and SKP2), and G2/mitotic-specific cyclin-B2 (CCNB2) genes (Figure 4D). Interestingly, TCGA data analysis revealed that the *CDK1*, *CDK2*, *CCNB2*, and *SKP2* genes were significantly upregulated, but the *SKP1* gene was downregulated in tumor samples compared with normal samples (Figure 4E). Knockdown experiments for CKS1B showed decreased gene expression levels of *CDK1*, *CDK2*, and *SKP2*, which have close functional interactions with CKS1B (Figure 4F). Taken together, the data indicate that CKS1B participates in the transcriptional regulation of an orchestrated set of genes that are associated with cell cycle progression and are thus linked to cancer cell proliferation and migration.

## 4. Discussion

CKS1B is a member of the cyclin kinase subunit 1 (CKS1) protein family and plays important roles in development and cell cycle regulation. However, it has been reported that CKS1B-overexpressing tumors display an increased cell cycle and malignant phenotypes through the activation of MEK1/2 and ERK1/2 phosphorylation [9,10]. Here, we also found that CKS1B was frequently upregulated in CRC tumor tissues compared with normal tissues and that a high level of CKS1B expression was associated with poor overall survival in CRC patients. Knockdown experiments for CKS1B showed significant cell growth and migration suppression, suggesting that CKS1B may function as an oncogene in CRC.

Normally, CKS1B interacts with cyclin and cyclin-dependent kinases (CDKs) to regulate the cell cycle. The CDK is a family of serine/threonine kinase and can regulate cell cycle progression and transcription. For example, the CDK1 interacts with cyclin B1 to form a heterodimer and maintains balance of cell survival and cell death during mitotic arrest. The CDK2 regulates progression through the S phase of cell cycle. The CDK2 and cyclin E activity plays a role in the cell cycle progression in mammalian cells [22]. However, several reports showed that upregulated CDKs and cyclins were frequently observed in many cancers, and their dysregulation resulted in deregulated mitosis and DNA replication [23,24,25]. In this context, it is not surprising to observe that expression of CDK1, CDK2, and cyclin B2 (CCNB2), which have close functional interactions with an oncogenic protein CKS1B, was significantly increased in colon cancer samples in the TCGA data set.

MicroRNAs are small noncoding RNA molecules that bind target mRNAs via complementary sequences in the 3’-UTR to inhibit their expression. MicroRNA dysregulation is associated with developmental disorders and the progression of cancers such as CRC. For example, miR-143, which is frequently downregulated in CRC, exerts tumor-suppressive functions by targeting KRAS oncogene expression [26]. On the other hand, upregulation of an oncogenic microRNA, miR-21, promoted CRC progression by downregulating various tumor suppressors, including phosphatase and tensin homolog (PTEN) [27]. miR-135b may also play a role in the early stage of CRC. It is increased in colon adenomas and negatively regulates the expression of the adenomatous polyposis coli (APC) gene [28]. Although miR-1258 has already been reported to act as a tumor suppressor by targeting heparanase in nonsmall cell lung cancer, breast cancer, and gastric cancer [29,30,31,32], its function in CRC is unclear. In this study, we found for the first time that miR-1258 plays a tumor-suppressive function in CRC, as its increased expression resulted in the downregulation of the oncogene CKS1B.

Previously, other groups identified that miR-197 and/or miR-204 negatively regulated CKS1B expression in non-small cell lung cancer and gastric cancer, respectively [33,34]. Using bioinformatics algorithms, we identified six candidate miRNAs that target CKS1B expression: miR-125a, miR-181a-1, miR-197, miR-361, miR-485, and miR-1258. Ectopic expression experiments revealed that CKS1B expression was decreased in only miR-1258 mimic-treated CRC cells (Figure 1C). On the other hand, although miR-197 has been reported to modulate CKS1B expression in nonsmall cell lung cancer, it did not affect CKS1B expression in CRC. These results indicated that microRNA-dependent modulation of oncogene expression may be context-specific and that miR-1258-dependent CKS1B regulation may be a specific event in CRC. miR-1258 overexpression reduced CRC cell proliferation and migration in vitro and tumorigenicity in vivo, similar to the results in CRC cells with CKS1B silencing. By contrast, treatment with miR-1258 inhibitor increased CRC cell proliferation and mobility in vitro. These findings further support the tumor-suppressive roles of miR-1258 in CRC.

Regulatory mechanisms involved in miR-1258 expression have not yet been clearly demonstrated in CRC. However, some mechanisms have been proposed after it was shown that miR-1258 downregulation was caused by DNA hypermethylation of the miR-1258 promoter region in prostate cancer and ovarian cancer; in those studies, a negative correlation between miR-1258 expression level and DNA methylation status was found [35,36]. Therefore, it was proposed that miR-1258 downregulation in CRC may be epigenetically regulated and that dysregulated miR-1258 may contribute to human CRC carcinogenesis. However, we did not report any confirmative data to support that in the present study.

The tumor-suppressive function of miR-1258 may apply to using miRNA mimic therapeutics for the treatment of CRC patients. Although miRNA therapeutics have no FDA-approved candidates, several drug candidates are already in clinical development or phase 1 or phase 2 clinical trials. For example, a phase 1 trial was completed for miR-16 (TargomiR), which is encouraging for patients with malignant pleural mesothelioma or non-small cell lung cancer. TargomiR contains a miRNA mimic, bacterially derived minicells and a targeting moiety. TargomiR uses miR-16 for its tumor-suppressive function and an EGFR antibody as a targeting moiety to consistently target deregulated lung cancer cells [37]. Therefore, using miR-1258 mimics and an appropriate targeting moiety (Frizzled receptors or K-Ras) can result in the development of new drugs and improve survival for CRC patients.

In the current study, we found that CKS1B was dramatically upregulated, but miR-1258 expression was downregulated in CRC patients. CKS1B expression was negatively regulated by miR-1258, which led to the suppression of cell proliferation, migration, and tumorigenicity in CRC cells, indicating that miR-1258 functions as a tumor suppressor in CRC. In conclusion, the precise modulation of miR-1258 expression downregulation in CRC patients might provide a potential therapeutic strategy to suppress CRC progression, although its therapeutic significance should be evaluated in the clinical setting.

## Figures and Tables

**Table 1:** Sequences.

Primers		Sequences (5′–3′)	Restriction Enzyme
CKS1B	Forward	TATTCGGACAAATACGACGACG	
	Reverse	CGCCAAGATTCCTCCATTCAGA	
GAPDH	Forward	CTCTGCTCCTCCTGTTCGAC	
	Reverse	TTAAAAGCAGCCCTGGTGAC	
miR-1258 cloning primer	Forward	AAAGAGCTCGAGCGTGAGCAACAGCTTC	SacI
	Reverse	AAGGTACCGGGACCTTCTCTCTGCTCCT	KpnI
CKS1B WT 3′-UTR	Forward	AGCTTTACACAGCTGTCCTTACTTCCTAACATCTT	SacI
	Reverse	TCGAAAGATGTTAGGAAGTAAGGACAGCTGTGTAAAGCTAGCT	XhoI
CKS1B MUT 3′-UTR	Forward	AGCTTTACACAGCTGTCCTTACTTAAATTTATCTT	SacI
	Reverse	TCGAAAGATAAATTTAAGTAAGGACAGCTGTGTAAAGCTAGCT	XhoI

## References

[1] Siegel R.L., Miller K.D., Jemal A. (2019). Cancer statistics, 2019. CA Cancer J. Clin..

[2] Luebeck E.G., Moolgavkar S.H. (2002). Multistage carcinogenesis and the incidence of colorectal cancer. Proc. Natl. Acad. Sci. USA.

[3] Hammond W.A., Swaika A., Mody K. (2016). Pharmacologic resistance in colorectal cancer: A review. Ther. Adv. Med. Oncol..

[4] Siegel R., Ma J., Zou Z., Jemal A. (2014). Cancer statistics, 2014. CA Cancer J. Clin..

[5] Hayles J., Beach D., Durkacz B., Nurse P. (1986). The fission yeast cell cycle control gene cdc2: Isolation of a sequence suc1 that suppresses cdc2 mutant function. Mol. Gen. Genet. MGG.

[6] Lee E.K., Kim D.G., Kim J.S., Yoon Y. (2011). Cell-cycle regulator Cks1 promotes hepatocellular carcinoma by supporting NF-kappaB-dependent expression of interleukin-8. Cancer Res..

[7] Ganoth D., Bornstein G., Ko T.K., Larsen B., Tyers M., Pagano M., Hershko A. (2001). The cell-cycle regulatory protein Cks1 is required for SCF (Skp2)-mediated ubiquitinylation of p27. Nat. Cell Biol..

[8] Spruck C., Strohmaier H., Watson M., Smith A.P., Ryan A., Krek T.W., Reed S.I. (2001). A CDK-independent function of mammalian Cks1: Targeting of SCF (Skp2) to the CDK inhibitor p27Kip1. Mol. Cell.

[9] Zhan F., Colla S., Wu X., Chen B., Stewart J.P., Kuehl W.M., Barlogie B., Shaughnessy J.D. (2007). CKS1B, overexpressed in aggressive disease, regulates multiple myeloma growth and survival through SKP2- and p27Kip1-dependent and -independent mechanisms. Blood.

[10] Kang Y.S., Jeong E.J., Seok H.J., Kim S.K., Hwang J.S., Choi M.L., Jo D.G., Kim Y., Choi J., Lee Y.J. (2019). Cks1 regulates human hepatocellular carcinoma cell progression through osteopontin expression. Biochem. Biophys. Res. Commun..

[11] Bartel D.P. (2004). MicroRNAs: Genomics, biogenesis, mechanism, and function. Cell.

[12] Doma M.K., Parker R. (2006). Endonucleolytic cleavage of eukaryotic mRNAs with stalls in translation elongation. Nature.

[13] Friedman R.C., Farh K.K., Burge C.B., Bartel D.P. (2009). Most mammalian mRNAs are conserved targets of microRNAs. Genome Res..

[14] Lau N.C., Lim L.P., Weinstein E.G., Bartel D.P. (2001). An abundant class of tiny RNAs with probable regulatory roles in Caenorhabditis elegans. Science.

[15] Lagos-Quintana M., Rauhut R., Lendeckel W., Tuschl T. (2001). Identification of novel genes coding for small expressed RNAs. Science.

[16] Esquela-Kerscher A., Slack F.J. (2006). Oncomirs—microRNAs with a role in cancer. Nat. Rev. Cancer.

[17] Han T.S., Hur K., Xu G., Choi B., Okugawa Y., Toiyama Y., Oshima H., Oshima M., Lee H.J., Kim V.N. (2015). MicroRNA-29c mediates initiation of gastric carcinogenesis by directly targeting ITGB1. Gut.

[18] Yan L.X., Wu Q.N., Zhang Y., Li Y.Y., Liao D.Z., Hou J.H., Fu J., Zeng M.S., Yun J.P., Wu Q.L. (2011). Knockdown of miR-21 in human breast cancer cell lines inhibits proliferation, in vitro migration and in vivo tumor growth. Breast Cancer Res..

[19] Lujambio A., Calin G.A., Villanueva A., Ropero S., Sanchez-Cespedes M., Blanco D., Montuenga L.M., Rossi S., Nicoloso M.S., Faller W.J. (2008). A microRNA DNA methylation signature for human cancer metastasis. Proc. Natl. Acad. Sci. USA.

[20] Han T.S., Ban H.S., Hur K., Cho H.S. (2018). The Epigenetic Regulation of HCC Metastasis. Int. J. Mol. Sci..

[21] Han T.S., Voon D.C., Oshima H., Nakayama M., Echizen K., Sakai E., Yong Z.W.E., Murakami K., Yu L., Minamoto T. (2018). Interleukin 1 Upregulates MicroRNA 135b to Promote Inflammation-associated Gastric Carcinogenesis in Mice. Gastroenterology.

[22] Malumbres M. (2014). Cyclin-dependent kinases. Genome Biol..

[23] Warfel N.A., Dolloff N.G., Dicker D.T., Malysz J., El-Deiry W.S. (2013). CDK1 stabilizes HIF-1alpha via direct phosphorylation of Ser668 to promote tumor growth. Cell Cycle.

[24] Sakurikar N., Thompson R., Montano R., Eastman A. (2016). A subset of cancer cell lines is acutely sensitive to the Chk1 inhibitor MK-8776 as monotherapy due to CDK2 activation in S phase. Oncotarget.

[25] Shubbar E., Kovacs A., Hajizadeh S., Parris T.Z., Nemes S., Gunnarsdottir K., Einbeigi Z., Karlsson P., Helou K. (2013). Elevated cyclin B2 expression in invasive breast carcinoma is associated with unfavorable clinical outcome. BMC Cancer.

[26] Chen X., Guo X., Zhang H., Xiang Y., Chen J., Yin Y., Cai X., Wang K., Wang G., Ba Y. (2009). Role of miR-143 targeting KRAS in colorectal tumorigenesis. Oncogene.

[27] Xiong B., Cheng Y., Ma L., Zhang C. (2013). MiR-21 regulates biological behavior through the PTEN/PI-3 K/Akt signaling pathway in human colorectal cancer cells. Int. J. Oncol..

[28] Nagel R., le Sage C., Diosdado B., van der Waal M., Oude Vrielink J.A., Bolijn A., Meijer G.A., Agami R. (2008). Regulation of the adenomatous polyposis coli gene by the miR-135 family in colorectal cancer. Cancer Res..

[29] Shi J., Chen P., Sun J., Song Y., Ma B., Gao P., Chen X., Wang Z. (2017). MicroRNA-1258: An invasion and metastasis regulator that targets heparanase in gastric cancer. Oncol. Lett..

[30] Tang D., Zhang Q., Zhao S., Wang J., Kangping L., Song Y., Zhao L., Kang X., Wang J., Xu S. (2013). The expression and clinical significance of microRNA-1258 and heparanase in human breast cancer. Clin. Biochem..

[31] Zhang L., Sullivan P.S., Goodman J.C., Gunaratne P.H., Marchetti D. (2011). MicroRNA-1258 suppresses breast cancer brain metastasis by targeting heparanase. Cancer Res..

[32] Liu H., Chen X., Gao W., Jiang G. (2012). The expression of heparanase and microRNA-1258 in human non-small cell lung cancer. Tumor Biol..

[33] Fujita Y., Yagishita S., Hagiwara K., Yoshioka Y., Kosaka N., Takeshita F., Fujiwara T., Tsuta K., Nokihara H., Tamura T. (2015). The clinical relevance of the miR-197/CKS1B/STAT3-mediated PD-L1 network in chemoresistant non-small-cell lung cancer. Mol. Ther..

[34] Shrestha S., Yang C.D., Hong H.C., Chou C.H., Tai C.S., Chiew M.Y., Chen W.L., Weng S.L., Chen C.C., Chang Y.A. (2017). Integrated MicroRNA-mRNA Analysis Reveals miR-204 Inhibits Cell Proliferation in Gastric Cancer by Targeting CKS1B, CXCL1 and GPRC5A. Int. J. Mol. Sci..

[35] Torres-Ferreira J., Ramalho-Carvalho J., Gomez A., Menezes F.D., Freitas R., Oliveira J., Antunes L., Bento M.J., Esteller M., Henrique R. (2017). MiR-193b promoter methylation accurately detects prostate cancer in urine sediments and miR-34b/c or miR-129-2 promoter methylation define subsets of clinically aggressive tumors. Mol. Cancer.

[36] Braga E.A., Loginov V.I., Burdennyi A.M., Filippova E.A., Pronina I.V., Kurevlev S.V., Kazubskaya T.P., Kushlinskii D.N., Utkin D.O., Ermilova V.D. (2018). Five Hypermethylated MicroRNA Genes as Potential Markers of Ovarian Cancer. Bull. Exp. Biol. Med..

[37] Reid G., Kao S.C., Pavlakis N., Brahmbhatt H., MacDiarmid J., Clarke S., Boyer M., van Zandwijk N. (2016). Clinical development of TargomiRs, a miRNA mimic-based treatment for patients with recurrent thoracic cancer. Epigenomics.

